# Abnormal Behaviors and Microstructural Changes in White Matter of Juvenile Mice Repeatedly Exposed to Amphetamine

**DOI:** 10.1155/2011/542896

**Published:** 2011-06-29

**Authors:** Hong-Ju Yang, Lijun Wang, Qiang Cheng, Haiyun Xu

**Affiliations:** ^1^Department of Anatomy, School of Medicine, Southern Illinois University Carbondale, 1135 Lincoln Drive, Carbondale, IL 62901, USA; ^2^Department of Computer Science, Southern Illinois University Carbondale, IL 62901-4328, USA

## Abstract

Amphetamine (AMP) is an addictive CNS stimulant and has been commonly abused by adolescents and young adults, during which period brain white matter is still developing. This study was to examine the effect of a nonneurotoxic AMP on the white matter of juvenile mice. *d*-AMP (1.0 mg/kg) was given to young male C57BL/6 mice once a day for 21 days. The spatial working memory and locomotion of mice were measured at the end. Then, mice were sacrificed and their brains were processed for morphological analyses to examine the white matter structure and for Western blot analysis to measure three main proteins expressed in mature oligodendrocytes. AMP-treated mice displayed higher locomotion and spatial working memory impairment and showed lower levels of Nogo-A and GST-pi proteins in frontal cortex and lower MBP protein in the frontal cortex and hippocampus. They also had fewer mature oligodendrocytes and weak MBP immunofluorescent staining in the same two brain regions. But the striatum was spared. These results suggest that the late-developing white matter is vulnerable to AMP treatment which is able to increase striatal and cortical dopamine. Both the compromised white matter and increased dopamine may contribute to the observed behavioral changes in AMP-treated mice.

## 1. Introduction

Amphetamine (AMP) is a drug approved by FDA for the treatment of attention-deficit/hyperactivity disorder (ADHD) and narcolepsy. This drug, similar to the others in the same group (amphetamines) consisting of AMP, methamphetamine, and methylenedioxymethamphetamine (MDMA), produces its principal effects by increasing synaptic levels of the biogenic amines, dopamine, norepinephrine, and serotonin through multiple mechanisms [1, 2]. While the therapeutic efficacy of AMP on ADHD has been appreciated, this drug is also an addictive CNS stimulant and has been used illegally. In fact, amphetamines are the prescription drugs most commonly abused by adolescents and young adults. Abuse of amphetamines constitutes a serious public health concern with worldwide abuse of them surpassing that of cocaine and opiates combined [3, 4]. 

It is well established that exposure of experimental animals to acute, high doses of amphetamines produces damage, generally referred to as “neurotoxicity,” to dopaminergic neurons innervating the dorsal striatum (caudate putamen) [4, 5]. Also, chronic intermittent administration of AMP to rodents produces a progressive and enduring increase in hyperactivity and stereotyped behavior [6–8]. This phenomenon, called behavioral sensitization, has been widely recognized as an animal model of lasting susceptibility to exacerbation of psychostimulant-induced psychosis [9]. Although these previous data are informative, the relevance of them to the consequences of low-dose, prescription use of amphetamines in humans is not clear. 

Over the past years, some human studies examined the white matter of amphetamines abusers. For example, Thompson et al. [10] found significant hypertrophy of the white matter in the methamphetamine abusers. After partitioning the white matter into lobes, the authors found that methamphetamine abusers had hypertrophy of the temporal white matter and occipital white matter. This white matter abnormality was thought to result from altered myelination and adaptive glial changes, including gliosis secondary to neuronal damage. Another white matter abnormality is white matter signal hyperintensities (WMH), which can be defined as patchy or diffuse white matter changes on T2-weighted magnetic resonance images (MRI) [11]. Methamphetamine abusers showed greater severity of WMH than the healthy subjects and severity of deep WMH correlated with total cumulative dose of methamphetamine [12]. More recent studies employed a new MRI technique, diffusion tensor imaging (DTI), to probe white matter microstructure with two useful indices: fractional anisotropy (FA) and mean diffusivity (MD). FA describes the directional variance of diffusional motion, and MD is an indicator of the overall magnitude of diffusional motion. Compared to healthy subjects, methamphetamine abusers showed significantly lower FA values in prefrontal white matter [13], genu of corpus callosum [14, 15], and mid-caudal superior corona radiata [16]. However, no information is available about effects of nonneurotoxic doses of amphetamines on white matter of animal brains.

On the basis of the aforementioned human studies, we hypothesized that chronic repeated administration of a nonneurotoxic dose of AMP could cause microstructural changes in white matter of mouse brain. To test this hypothesis, this study took advantage of juvenile C57BL/6 mice during the process of myelination, which continues to progress through adolescence in rodents [17]. The protracted time course for this process has been demonstrated to be vulnerable to perturbation in both rodents and humans [18–22]. We administered *d*-AMP once daily to C57BL/6 mice for three weeks. The spatial working memory of mice was measured two days after the last injection and the locomotion five days. After finishing behavioral tests, mice were sacrificed and their brains were processed for morphological analyses to examine the micro-structure of white matter, which is one of the two components of the central nervous system (CNS) and consists mostly of myelinated axons. The myelin sheath of myelinated axons is produced by oligodendrocytes located in both grey and white matter. The myelinated axons can be examined in brain sections immunohistochemically stained using the specific antibody to the myelin basic protein (MBP) believed to be a marker of myelin sheath. The oligodendrocytes can be labeled by specific antibodies to the pi form of glutathione-stransferase (GST-pi) or Nogo-A, two proteins expressed in mature oligodendrocytes In addition, Western blot analysis was performed to measure the amounts of all above-mentioned proteins of tissue samples from the brain regions of the frontal cortex, hippocampus, and striatum.

## 2. Materials and Methods

### 2.1. Animals and Treatments

All animal procedures in this study were in accordance with the National Institute of Health Guide for the Care and Use of Laboratory Animals and were approved by the Animal Care and Use Committee of Southern Illinois University at Carbondale.

Twenty-four male juvenile (postnatal day 28, P28) C57BL/6 mice (Charles River Laboratories, Wilmington, Mass, USA) were used in this study. They were kept in Plexiglas cages with free access to food and water, in a room with controlled temperature (22 ± 1°C) and on a 12 h light/dark cycle (lights on at 07:00 h). After one week acclimatization, the mice were randomly assigned to two groups of twelve each. Mice in the control group (CNT) received intraperitoneal (i.p.) injection of 0.9% saline (10 mL/kg) once a day for 3 weeks. During the same period (P36–56), mice in the experiment group (AMP) received i.p. injection of *d*-AMP sulphate (1.0 mg/kg, dissolved in saline) once a day (Sigma-Aldrich, St. Louis, MO, USA). This dosage is much lower than the neurotoxic doses (≥15 mg/kg, continuous systemic administration) in nonhuman studies [23] and may be comparable to the standard AMP treatment for core ADHD symptoms (0.1 to 0.5 mg/kg, oral administration), given the much higher basal metabolic rate in rodents than in humans [24]. The period P36–56 corresponds to puberty or adolescence in humans when myelination continues slowly at least in the prefrontal cortex [25, 26]. The AMP solution was prepared freshly and given to mice based on their body weight which was measured every day. 

### 2.2. Y-Maze Test

The Y-maze test, a simple recognition test of measuring spatial working memory of rodents [27–29], was performed 24 hours after the last AMP/saline administration. Each mouse was placed at the end of one arm of a symmetrical Y-maze (15 cm of two shorter arms and 20 cm of the longer arm) and allowed to move freely through the maze for an 8-min test period. Alternation was defined as successive entries into the three arms on overlapping triplet sets. The maximum number of possible spontaneous alternations was determined as the total number of arms entered-2, and the percentage was calculated as the ratio of actual to possible alternations × 100.

### 2.3. Open-Field Test

The open-field was performed five days after the last AMP/saline administration. The open-field apparatus used in this study was a square wooden box (56 cm × 56 cm × 31 cm) with an open top and floor divided into 9 equal smaller squares (units). Fifteen minutes after a challenge injection of AMP (0.5 mg/kg; given to mice in both the control and AMP groups), a mouse was placed in the center of the open-field arena and allowed to move freely around the open field for 15 min. The moving path of the mouse was recorded by a video camera, which was placed above the arena and connected to the computer installed with the video tracking program SMART (San Diego Instruments, Calif, USA). Specifically, the following parameters were analyzed and compared between the groups in the present study.

Total ambulation frequency = number of any floor unit entered;

Peripheral squares ambulation frequency = number of entrances into the floor units close to the walls of the apparatus;

Central square ambulation frequency = number of entrances into the central unit of the apparatus.

### 2.4. Western Blot Analysis

Mice (*n* = 6 per group) were sacrificed under deep anesthesia. Their brains were rapidly removed; then the frontal cortex, hippocampus, and striatum were dissected out and stored at −80°C until use. Western Blot analysis was performed to assess the amount of MBP, GST-pi, and Nogo-A proteins in each brain region. Briefly, equal amounts of protein (10–30 *μ*g) was separated by 12.5% (for MBP and GST-pi) or 8% (for Nogo-A) SDS-polyacrylamide gel electrophoresis and then transferred to polyvinylidene difluoride membranes in a Tris-glycine buffer with 20% methanol using a power of 20 V overnight. Membranes were then blocked in 5% skim milk in TBST (10 mM Tris, pH 7.5, 150 mM NaCl plus 0.05% Tween-20) and incubated overnight at 4°C with their primary antibodies diluted in TBST. The antibodies used were rabbit anti-NogoA (1 : 1000, Sigma-Aldrich, MO, USA), rabbit anti-MBP (1 : 4000, Chemicon, Calif, USA), rabbit anti-GST-pi (1 : 1000, Stressgen, Canada), and rabbit anti-*β*-actin (1 : 2000; Sigma-Aldrich, MO, USA). They were then washed three times in TBST and subsequently incubated in TBST buffer containing secondary antibody (Alexa Fluor 680, invitrogen, Calif, USA). Image was scanned with ODYSSEY (Li-COR, Biosciences) and analyzed with Image J software (NIH, Bethesda, MD, USA). Values of proteins of interest were normalized to *β*-actin, and the ratios were then used to perform statistical analysis.

### 2.5. Immunofluorescence

Animals (*n* = 6 per group) were deeply anesthetized with chloral hydrate (400 mg/kg i.p.) and perfused through the ascending aorta with 0.1 M phosphate buffered saline (PBS; pH 7.4), followed by 4% paraformaldehyde in PBS. Their brains were then removed and immersed in the same fixative overnight, followed by cryoprotection in 25% sucrose at 4°C for 24–48 h. Serial coronal sections (20 *μ*m) of the brains were cut using a sliding microtome (Lipshaw, Detroit, Mich, USA) and collected in six well plates containing 0.01-M PBS. Free-floating sections were incubated in 1% H_2_O_2_/10% Triton X-100 in PBS for 10 min, and then washed with PBS. Sections were incubated overnight with anti-MBP (MAB 386; 1 : 200, Chemicon, Temecula, Cailf, USA) or anti-GST-pi (1 : 200, Stressgen, Canada). FITC-conjugated donkey anti-rabbit IgG (1 : 100; Chemicon, Temecula, Cailf, USA) visualized the GST-pi epitope and Rhodamine-conjugated donkey antimouse IgG visualized the MBP epitope. Controls obtained by omitting the primary antibody were run for each immunofluorescent staining. Images of stained mouse brain sections were acquired with a DMI 4000 B microscope (Leica, German) equipped with a digital capture system. Image-Pro Plus software (Version 6.0, Media Cybernetics, Inc., Bethesda, MD, USA) was used to count the number of GST-pi positive cells in the cerebral cortex (including frontal cortex and parietal cortex) and corpus callosum and to quantify pixel intensity values of MBP staining in the cerebral cortex, corpus callosum, and hippocampus. The values of the AMP group were normalized as the percentages of those in CNT group.

### 2.6. Immunohistochemistry

Free floating sections were pretreated with 0.5% hydrogen peroxide in methanol for 20 min, then washed with PBS, and incubated for 1 h at 22°C with a blocking solution composed of 0.2% Triton X-100 and 5% normal rabbit or goat serum in PBS. Sections were then incubated with rabbit anti-Nogo-A (1 : 100; Sigma-Aldrich, MO) in the blocking solution for 48 h at 4°C. After rinsed in PBS, sections were incubated in biotinylated secondary antiserum (1 : 200) of the Vectastain (Elite) ABC kits (Burlingame, Calif, USA) for 2 h at 22°C. Following PBS rinses, the sections were incubated in an avidin-biotin-horseradish peroxidase complex (ABC) for 1 h at 22°C. Then the antigen antibody complexes were visualized using 0.025% 3,3′-diaminobenzidine (DAB, Sigma-Aldrich, St. Louis, MO, USA) as the chromogen. The immunohistochemically stained sections were examined under the DMI 4000 B microscope. The numbers of Nogo-A positive cells in the cerebral cortex (including frontal cortex and parietal cortex) and corpus callosum were counted using the Image-Pro Plus software. 

The values of the AMP group were normalized as the percentages of those in CNT group.

### 2.7. Data Analysis

The data from Y-maze test and Western blot analysis were analyzed by *Student t*-test. Each Western blot test had samples from a same brain region of mice in CNT and AMP groups therefore, brain region was not considered as a factor (data from different brain regions were not combined). The other data were analyzed by two-way analysis of variance (ANOVA), followed by *Post hoc* comparisons of the Newman-Keuls test. When a *P*  value was less than  0.05, the difference was considered significant.

## 3. Results

### 3.1. Behavioral Changes in AMP-Treated Mice

**Figure 1:**
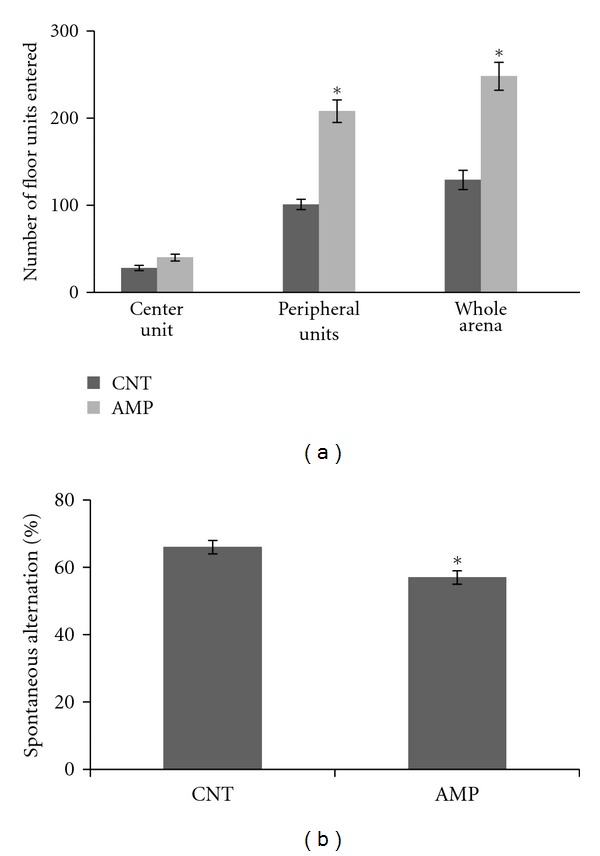
AMP-induced behavioral changes. Mice were administered intraperitoneally with 1.0 mg/kg d-AMP once a day for 21 days or a same volume of saline during the same period. The spatial working memory of mice was measured two days after the last injection and the locomotion five days. Data were expressed as means ± SEM. Comparisons were made between CNT and AMP groups. *P < 0.05. For open-field test, all 12 mice in the AMP group entered the peripheral units more often than the average of CNT group. For Y-maze test, of 12 subjects in the AMP group, two mice showed spontaneous alternations (68 and 71%) above the average of CNT group (66%).

For open-field test, Two-way ANOVA analysis was performed and the result indicated a significant effect of areas (central, peripheral, and whole arena) on the numbers of floor units entered (*F*_  5,71_ = 116; *P* < 0.0001). *Post hoc* comparisons suggested that AMP-treated mice had more locomotion in the peripheral zone of the open-field arena than the controls; the total locomotion of AMP-treated mice was also higher. But the difference in their locomotion in the center zone did not reach a significant level (*P* = 0.07) (Figure 1(a)). For Y-maze test, *Student* *t*-test indicated that the AMP-treated mice had decreased spontaneous alternation compared to controls that had never exposed to AMP (Figure 1(b)). The numbers of arm entrance of the two groups were comparable (not shown).

### 3.2. Decreased Nogo-A, GST-pi, and MBP Proteins in AMP-Treated Mice

**Figure 2:**
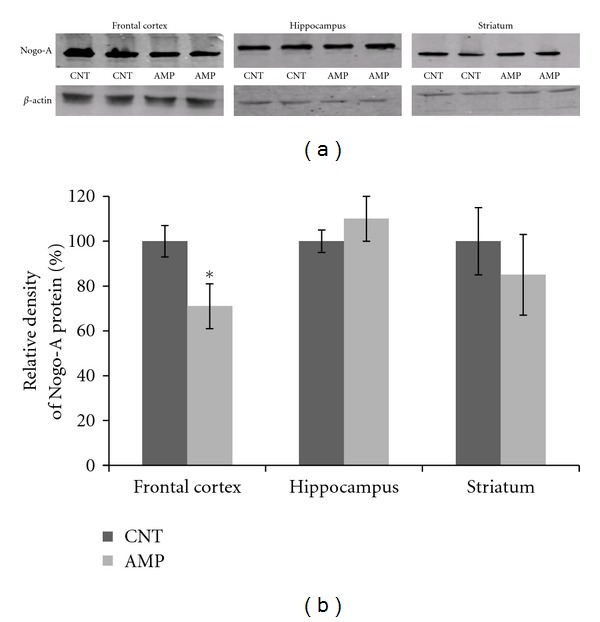
AMP reduced the amount of Nogo-A protein in the frontal cortex. After treating with d-AMP or a same volume of saline for 21 days and subjected to behavioral tests, mice were sacrificed, their brains were dissected, and tissue samples from specific brain regions were processed for Western blot analysis. The amount of Nogo-A protein in the frontal cortex, hippocampus, and striatum was measured. (a) shows representative Western blot images and (b) is a bar chart illustrating the statistical analysis of the Western blot results. Data were expressed as means ± SEM. Comparisons were made between CNT and AMP groups. *P < 0.05. Of 6 PFC samples in AMP group, one showed a ratio (1.5) of Nogo-A protein/*β*-actin higher than the average of CNT group (1.4).

**Figure 3:**
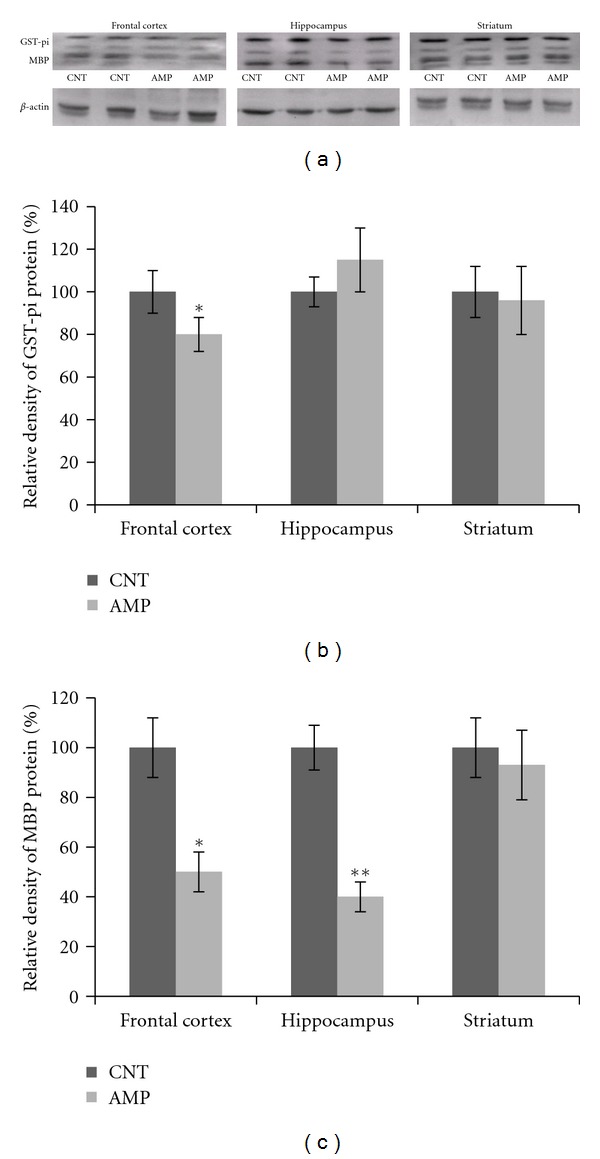
AMP reduced the amounts of GST-pi and MBP proteins in the frontal cortex and hippocampus. After treating with d-AMP or a same volume of saline for 21 days and subjected to behavioral tests, mice were sacrificed, their brains were dissected, and tissue samples from specific brain regions were processed for Western blot analysis. The amounts of GST-pi and MBP proteins in the frontal cortex, hippocampus, and striatum were measured. (a) shows representative Western blot images and (b) and (c) consists of two bar charts illustrating the statistical analysis of the Western blot results. Data were expressed as means ± SEM. Comparisons were made between CNT and AMP groups. *P < 0.05; **P < 0.01. In the measurement of GST-pi protein, all 6 frontal cortex samples in the AMP group showed lower levels than the average of CNT group. In the measurement of MBP protein, all frontal cortex and hippocampus samples in the AMP group showed lower levels than the average of CNT group.

Compared to the CNT group, AMP-treated mice showed a lower amount of Nogo-A in the frontal cortex but not in the hippocampus and striatum (Figure 2). Similarly, the AMP-treated mice had a lower level of GST-pi in the frontal cortex than the CNT group. In the same Western blot analysis, the AMP-treated mice showed lower levels of MBP protein in their frontal cortex and hippocampus but not in the striatum (Figure 3).

### 3.3. Decreased Mature Oligodendrocytes and MBP Staining in AMP-Treated Mice

**Figure 4:**
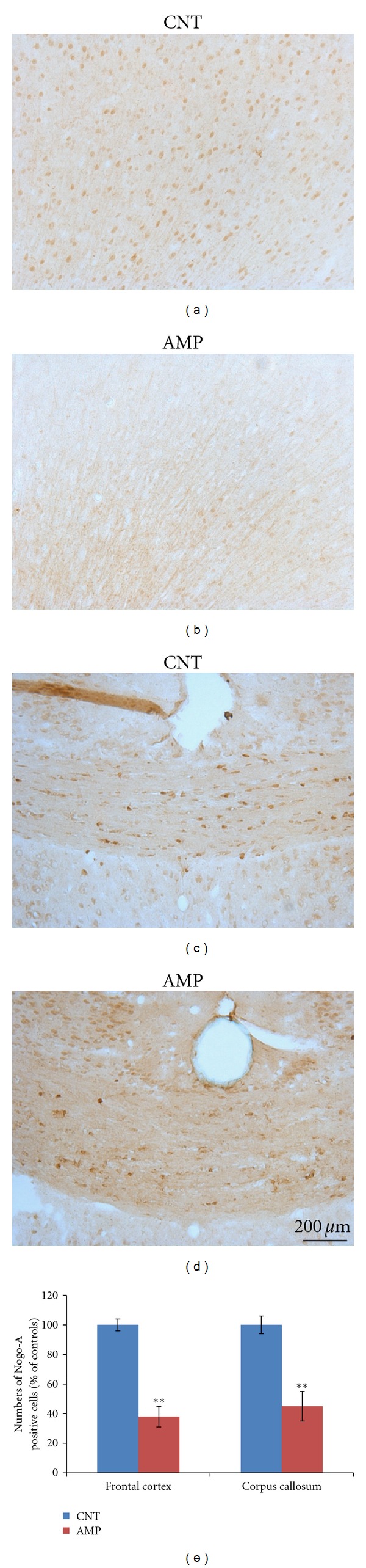
AMP reduced the numbers of Nogo-A positive cells in cerebral cortex and corpus callosum. After treating with d-AMP or a same volume of saline for 21 days and subjected to behavioral tests, mice were sacrificed and their brains were processed for immunohistochemical staining. The numbers of Nogo-A positive cells in cerebral cortex and corpus callosum were counted. All AMP-treated mice had decreased Nogo-A cells than the average of CNT group. Representative photographs were presented and labeled. Quantitative data were statistically analyzed and presented in a bar chart. Data were expressed as means ± SEM. Comparisons were made between CNT and AMP groups. **P < 0.01.

**Figure 5:**
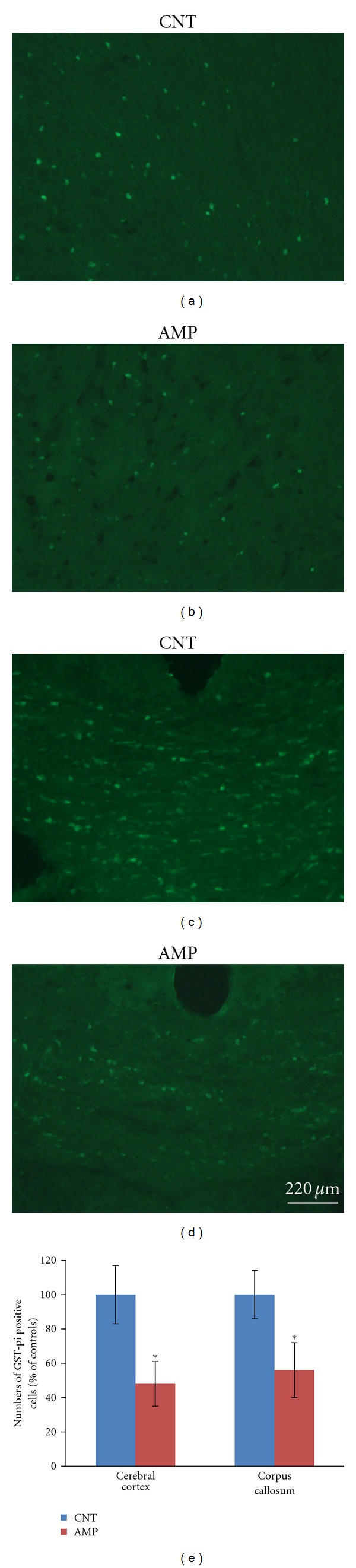
AMP reduced the numbers of GST-pi positive cells in cerebral cortex and corpus callosum. After treating with d-AMP or a same volume of saline for 21 days and subjected to behavioral tests, mice were sacrificed and their brains were processed for immune-fluorescent staining. The numbers of GST-pi positive cells in cerebral cortex and corpus callosum were counted. All AMP-treated mice had decreased GST-pi cells than the average of CNT group. Representative photographs were presented and labeled. Quantitative data were statistically analyzed and presented in a bar chart. Data were expressed as means ± SEM. Comparisons were made between CNT and AMP groups. *P < 0.05.

In brain sections, mature oligodendrocytes were labeled by the specific antibodies to Nogo-A or GST-pi and counted using the software Image-Pro Plus. The Nogo-A positive cells were in brown color as shown in Figure 4. Two-way ANOVA analysis was performed and the result indicated a significant treatment (AMP) effect on the number of Nogo-A positive cells in the brain regions (*F*_3,23_ = 113; *P* < 0.0001). Compared to the CNT group, the AMP-treated mice showed fewer Nogo-A positive cells in their cerebral cortex and corpus callosum (Figure 4). The GST-pi positive cells were labeled with green fluorescence and counted. Two-way ANOVA analysis was performed, and the result indicated a significant treatment (AMP) effect on the number of GST-pi positive cells in the brain regions (*F*_3,  23_ = 86; *P* < 0.0001). The cerebral cortex and corpus callosum of the AMP-treated mice showed fewer numbers of GST-pi positive cells than the CNT group (Figure 5). 

**Figure 6:**
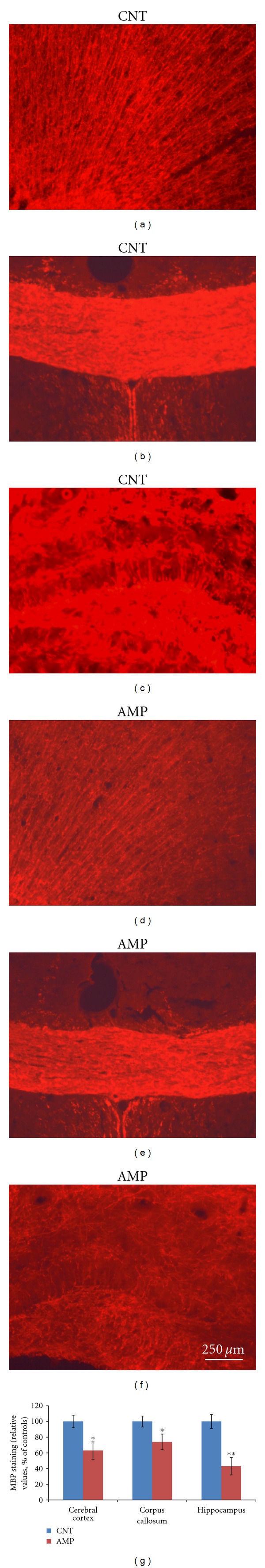
AMP reduced the MBP immune-fluorescent staining in cerebral cortex, corpus callosum and hippocampus. After treating with d-AMP or a same volume of saline for 21 days and subjected to behavioral tests, mice were sacrificed, and their brains were processed for MBP immune-fluorescent staining. Representative photographs were presented and labeled. Quantitative data were statistically analyzed and presented in a bar chart. All AMP-treated mice had decreased values of MBP staining than the average of CNT group. Data were expressed as means ± SEM. Comparisons were made between CNT and AMP groups. *P < 0.05; **P < 0.01.

The MBP containing myelinated fibers were labeled with red color as shown in Figure 6 and the optical density of it was measured using the software Image-Pro Plus. Two-way ANOVA analysis was performed, and the result indicated a significant treatment (AMP) effect on the intensity of MBP immune-fluorescent staining (*F*_3,  23_ = 10.46; *P* < 0.01). The AMP-treated mice showed much weaker MBP staining in the cerebral cortex, corpus callosum, and hippocampus, as compared to the CNT group (Figure 6).

## 4. Discussion

Chronic repeated administration of 1.0 mg/kg AMP to juvenile mice induced a behavioral sensitization and impaired the spatial working memory of subjects (Figure 1); in the meanwhile, this treatment caused microstructural changes in white matter of the subjects (Figures 4–6). This dosage is much lower than the neurotoxic doses in nonhuman studies [23] and lower than those used in most of rodent studies investigating the behavioral sensitization effect of this drug [30–35]. Therefore, this treatment regimen was not expected to be of “neurotoxicity”. In fact, the averaged body weight changes of saline and AMP-treated mice during the experimental period overlapped perfectly (not shown). These results suggest that white matter is very vulnerable to AMP and that the microstructural changes in white matter of some brain regions contribute to the above-mentioned behavioral changes. These two suggestions have specific relevance to the uses of chronic low-dose AMP in adolescents and young adults considering the following conditions. First, in many cases AMP treatment of ADHD has been extended to adulthood from the initiation of it in adolescence as the disorder can persist into adulthood, with adverse occupational, academic, and interpersonal consequences [23, 36]; second, in newly diagnosed adults higher doses (0.9 mg/kg/day) are usually used for maximal benefit [37, 38]. Third, there is increasing illicit use of AMP among college students, who may not only take the drug orally but also dissolve and inject it [39]. Therefore, it should be aware of that chronic low-dose AMP may suppress the myelination process in brains of adolescents and young adults and thus causing behavioral problems.

Although high doses of AMP, comparable to amounts used by addicts, were shown to damage dopaminergic pathways [23], neurotoxicity was not usually seen in rats after administration of lower amounts, either by continuous exposure, when the doses were less than about 15 to 20 mg/kg/day, or when the exposure lasted for 3 days or less [40]. Therefore, the microstructural changes in the white matter of mouse brain by a nontoxic AMP treatment regimen in the present study suggest that white matter is more susceptible to AMP than the grey matter. This finding is interesting but not surprising, since that white matter undergoes a much longer postnatal developmental period than the grey matter does. In humans, the white matter volume increases across the adolescent years, particularly in frontoparietal regions [25, 26, 41]. Also, the micro-structure of white matter undergoes developmental changes during adolescence. For example, studies of typically developing adolescents showed increases in FA (high FA reflects greater fiber organization and coherence, myelination, and/or other structural components of the axon) and decreases in MD (low MD values suggest greater white matter density). These trends continue through early adulthood [42–46]. 

Most of the studies employing repeated administration of AMP generally investigated the effects of this regimen on locomotor activity and stereotypes and attributed AMP-induced higher locomotor activity to increased striatal and cortical dopamine [6–8]. However, only a few previous studies [31, 47] assessed possible deficits in learning and memory of AMP-treated mice. In the study by Mandillo et al. [31], 5 days of repeated injections (i.p.) of AMP (2.5 and 5.0 mg/kg per day) impaired the ability of mice to a spatial change when tested on day 6. In the study by Stefani and Moghaddam [47], rats administered AMP (2.5 mg/kg, twice daily) for 5 consecutive days showed impaired alternation performance relative to their pretreatment baseline score. In the present study, mice administered 1.0 mg/kg AMP (once a day) for 21 days showed impaired spontaneous alternation in the Y-maze (Figure 1). These experimental results may have relevance to schizophrenia, in which dysfunctional dopamine systems are fundamental, the impairment of cognitive functions has been recognized as a core feature, and the cognitive impairment has been correlated with white matter abnormalities [48, 49]. 

Low doses (0.15 and 0.25 mg/kg) of AMP were reported to increase synaptic and extracellular dopamine in prefrontal cortex of rat [50], and therapeutic doses of AMP increase synaptic and extracellular dopamine in the striatum [51]. On the basis of these previous results, it is plausible to suggest that the AMP treatment regimen in the present study could increase striatal and cortical dopamine. The increased dopamine activity in frontal cortex might contribute to spatial working memory impairment. In line with this speculation, patients with schizophrenia have impairment of cognitive functions as discussed before. In animal studies [52, 53], high level dopamine in frontal cortex was accompanied by impaired spatial working memory in mice, and this impairment was prevented by antipsychotic drugs. In addition, high levels of dopamine were reported to be toxic via the formation of oxidative metabolites resulting from dopamine degradation [54], which may kill oligodendrocytes and cause demyelination. In support of this speculation, high levels of dopamine in frontal cortex were seen to precede the demyelination in the same brain region of mice exposed to cuprizone [52], a copper chelator that was shown to induce demyelination [55]. Taken together, we suggest that the AMP-induced dopamine increases contribute to both impaired spatial working memory and the microstructural changes in white matter of mice. 

It is noteworthy that no changes were found in the striatum white matter, whereas changes were significant in cerebral cortex, corpus callosum, and hippocampus. This regional difference may be explained as follows. First, it is known that the baseline of dopamine concentration in striatum is the highest among the brain regions. This feature may make the stratum most tolerant to dopamine increase. Therefore, we may speculate that the AMP-induced increases in striatal dopamine might be tolerable in this study, thus did not damage white matter in the brain region. Second, it is also known that the frontal cortex and hippocampus develop later than the striatum. These late-developing brain regions are expected to be more susceptible to the AMP-induced increases in dopamine compared to the early-developed striatum. In line with this explanation, the frontal cortex, corpus callosum, and hippocampus were demonstrated to be most susceptible to cuprizone-induced demyelination [56], where dopamine increases preceded demyelination [52]. 

In summary, repeated administration of 1.0 mg/kg AMP increased the locomotion, impaired the spatial working memory, decreased protein levels of Nogo-A and GST-pi in the frontal cortex, reduced numbers of mature oligodendrocytes in the frontal cortex and corpus callosum, and lowered MBP protein in the frontal cortex and hippocampus of mice. The striatum was spared from this treatment regimen. These results suggest that the late-developing white matter of frontal cortex and hippocampus is very vulnerable to AMP, treatment which is able to increase striatal and cortical dopamine. Both compromised white matter and increased dopamine in these brain regions may contribute to increased locomotor activity and impaired spatial working memory in the AMP-treated mice.
